# Breaking our daily “ultra-processed” bread

**DOI:** 10.1371/journal.pmed.1004437

**Published:** 2024-07-25

**Authors:** Giles S. H. Yeo

**Affiliations:** MRC Metabolic Diseases Unit, Institute of Metabolic Science, Addenbrooke’s Hospital, University of Cambridge, Cambridge, United Kingdom

## Abstract

In the Editorial, Giles Yeo discusses if it is possible that the UPF concept could be doing more harm than good.

UPFs are the new bad kid on the block when it comes to diet and health. But what are they and is it possible that the UPF concept could be doing more harm than good?

The fields of dietary and nutritional science have captured the public’s imagination. We are experts at eating after all, knowing how much and when to eat, at least sufficiently well to have survived long enough to be reading this piece. The flipside to all of this public interest is that nearly everyone has an opinion, and in this world of social media, most are unafraid to express it. It is therefore a very noisy field in which to work, full of waxing and waning dietary fads, with foods once bad now thought to be good, or good now bad, and even good and bad at the same time! Which brings us to the hottest topic *du jour*, “ultra-processed” foods or UPFs.

The term UPF is derived from the NO*VA* (a name and not an acronym) system of food classification, proposed by Monteiro and colleagues [1]. Briefly, NO*VA* divides foods into 4 different groups. Unprocessed or minimally processed foods (group 1) include obviously unprocessed foods such as fresh fruits and vegetables, fresh or frozen meat and fish, and eggs. This group also includes foods that have been minimally processed, for example, by drying, grinding, pasteurisation, refrigeration, freezing, vacuum packaging, or nonalcoholic fermentation. Processed culinary ingredients (group 2) encompass processed ingredients extracted either directly from group 1 foods or from nature (sea-water, honey combs, or tree sap) by processes such as pressing, refining, grinding, milling, and drying. These group 2 ingredients tend to be used to prepare, season, and cook group 1 foods.

More extensive levels of processing shift foods into groups 3 and 4. Processed foods (group 3) can be made by adding sugar, oil, salt, or other group 2 substances to group 1 foods for instance. Processes include various preservation or cooking methods, and, in the case of breads and cheese, nonalcoholic fermentation. Everything else falls under the umbrella of ultra-processed food and drink products (group 4). As defined by Monteiro, UPFs are, in effect, industrial formulations, typically with 5 or usually many more ingredients, that most would find difficult, if not impossible, to replicate in a domestic situation.

What exactly is it about UPFs that makes it so apparently bad for us? First, because of the degree of processing, most UPFs are inherently lower in protein, fibre, or both. This makes UPFs very calorically available, meaning that they are easily digestible, and our body can extract a large proportion of the calories in the foods. Second, the high degree of processing also strips out much of the flavour, which then has to be replaced for the foods to be made palatable; thus, UPFs often contain higher amounts sugar, salt, and fat. Third, and most controversially, some argue that it is the processing that is inherently bad; the fact that many of the foods are so highly engineered. In truth, the evidence supporting this third reason is equivocal at best, and entirely absent at worst.

On the face of it, there is ample evidence that consumption of high volumes of UPFs is linked to poorer health outcomes, exemplified by the recent umbrella review of 45 different studies encompassing nearly 10 million people [2]. The authors reported direct associations between exposure to UPFs and 32 health parameters spanning mortality, cancer, mental health, respiratory, cardiovascular, gastrointestinal, and metabolic health outcomes.

There are, however, a couple of points to reflect on. First, the umbrella review rated the quality of evidence as low to very low for nearly all outcomes, and thus its conclusions need to be considered in that light. Second, we need to seriously consider if all UPFs are created equal. For example, there are foods that appear to be group 1 or 3 foods, but because they contain additives, become classed as group 4. So plain yoghurt is a group 1 food; however, if flavourings, sugar, artificial sweeteners, or sweetened granola are added, it becomes ultra-processed and moves into group 4. Bread is a processed food, but if made with added emulsifiers and gluten, it becomes ultra-processed. Surely, there are many others who feel that the imprecision of the term UPF is an issue?!

The biggest methodological weakness in dietary and nutritional science is the difficulty to conduct truly blinded randomised control trials (RCTs). Unlike in trials of drugs and other purified compounds that allow for properly blinded placebo controls, it is impossible to do this with whole foods. Thus, the vast majority of dietary studies are observational. Ultimately, the success of such studies depends on power and a precise measure, both of intake and outcome. UPFs encompass such a broad range of foods, some of which we should certainly be eating much less of; but others, including the aforementioned supermarket bread that sneak into group 4 with just the addition of emulsifiers and few other additives. Thus, many studies that use the NOVA criteria are reporting that UPFs, on average, are linked to poorer health outcomes. Which of the literally thousands of types of UPFs are truly “bad” for you, and which are harmless foods that have hitchhiked along through some quirk of nomenclature?

These questions are more than an esoteric academic exercise and have real world consequences. Returning to bread, while some of us can choose to stroll down our leafy suburban streets to purchase freshly baked artisanal sourdough, the cost of such a luxury item is prohibitive to many in our society. For example, the Food Foundation found that in January 2024, 15% of UK households, or around 8 million adults and 3 million children [3], were living in food insecurity, with similarly shocking numbers emerging from around the world [4]. Ultimately, all bread is primarily made from flour, salt, water, and yeast. Taste aside, is supermarket bread worse for you than artisanal bread?

The vast burden of noncommunicable diseases faced by humankind today is diet related [5], and there are certainly many foods that we undoubtedly should eat a lot less of. However, the UPF concept may be too imprecise to serve as an arbiter of how healthy or unhealthy a particular food might be, thus risking the demonisation of perfectly healthy and affordable foods in a time of soaring food insecurity.
